# Medial Tibial Stress Syndrome (Shin Splint): Prevalence, Causes, Prevention, and Management in Saudi Arabia

**DOI:** 10.7759/cureus.59441

**Published:** 2024-05-01

**Authors:** Majdi Hashim, Faisal A Alhazani, Ayedh S AlQarni, Mazen A Albogami, Abdulrahman O Alomar, Abdullah S Alsultan

**Affiliations:** 1 Department of Orthopedics, College of Medicine, Imam Mohammad Ibn Saud Islamic University (IMSIU), Riyadh, SAU; 2 College of Medicine, Imam Mohammad Ibn Saud Islamic University (IMSIU), Riyadh, SAU

**Keywords:** distal tibia, athletic leg pain, exertional leg pain, medial tibial stress syndrome, shin splints

## Abstract

Background/aims

Medial tibial stress syndrome (MTSS), also known as “shin splint”, is most often described as exertional leg pain along the shinbone (tibia), which occurs due to the inflammation of the muscles, tendons, and bone tissue in this area. This study aims to assess the prevalence, risk factors, and their association with the development of MTSS, as well as the effective treatments that reduce pain and improve functions among the Saudi general population.

Materials and method

The present cross-sectional study was conducted on the general population of Saudi Arabia through an electronic survey over a period of three months. The study sample of 443 patients was deemed and considered. The study included participants from the general population in Saudi Arabia above the age of 18. A structured self-response questionnaire was given to the participants after institutional research ethical approval was obtained for the study.

Results

Among the 443 participants, the majority were male (n = 228, 51.5%), aged 18-29 (n = 227, 51.2%), and residing in the central region of Saudi Arabia (n = 398, 89.8%). Most participants reported engaging in sporting activities (n = 211, 47.6%), with high-intensity training being the most common (n = 93, 44.1%). Only a small proportion (n = 8, 1.8%) reported a previous diagnosis of MTSS. Analysis revealed associations between MTSS prevalence and certain demographic factors, including walking surface preferences and engagement in specific sports. Treatment strategies for MTSS included rest, ice application, physiotherapy, and pain-relieving medication, with varying degrees of satisfaction and recurrence rates among participants.

Conclusion

The study provides valuable insights into the prevalence, risk factors, management, and preventive measures related to MTSS among the Saudi general population. While certain demographic factors and exercise practices were associated with MTSS prevalence, effective treatment options such as rest, physiotherapy, and appropriate footwear were reported. Moreover, adherence to preventive measures such as stretching, proper footwear selection, and gradual training progression may help mitigate the risk of MTSS development.

## Introduction

Medial tibial stress syndrome (MTSS), also known as “shin splint”, is most often described as exertional leg pain along the shinbone (tibia), which occurs due to the inflammation of the muscles, tendons, and bone tissue in this area [1]. Shin splints affect between 13.6% and 20% of runners and contribute to up to 60% of lower limb injuries [2]. Individuals with MTSS frequently have bilateral pain or discomfort on the medial side of the tibia, most frequently in the distal region. The discomfort is normally worse the next day, but it will subside gradually, commonly felt in the middle of the medial tibia. However, it can impact the entire length of the leg. In cases of severe and chronic MTSS [3], pain may be felt even at rest. Shin splints were seen in more females (55.3%) than males (44.7%) [4]. Training on hard surfaces or uneven surfaces, as well as footwear changes, are etiological factors in the development of shin splints [5]. Moreover, military recruits and runners are all at risk of shin splints. Those who have recently increased or adjusted their training routines, as well as those who have not adequately warmed up or have hyper-pronation of the foot, are prone to MTSS. This type of injury can interfere with an athlete's performance and all of their activities [6]. Runners who had previously sustained a running injury were more than twice as likely to experience an MTSS [7]. Individuals with a higher BMI and those who had previously worn orthotic devices for an extended period of time were shown to be more prone to developing MTSS [8]. Additionally, people with vitamin D insufficiency, osteoporosis, and flat feet are more likely to get shin splints [9]. During running, heel striking induces fast internal rotation of the tibia as the calcaneus transitions from an inverted to an everted position, resulting in traction periostitis of the soleus or flexor digitorum longus muscles, bending, and bowing of the tibia. Excessive pronation puts more strain on the posterior tibial tendon, slowing the internal rotation of the tibia and lowering the arch [5]. In another study, MTSS was associated with decreased hip internal rotation and increased ankle plantar flexion [10]. A study also found no link between the deep crural fascia, soleus, flexor digitorum longus, and tibialis at the posterior border of the tibialis, where pain showed as MTSS. Furthermore, a navicular drop was assessed with feet shoulder-width apart, and it was revealed to be significantly associated with MTSS [8]. Several investigations found reduced tibial bone mineral densities in the MTSS region, which improved to normal levels once the MTSS symptoms ceased [11,12]. Another study discovered microcracks without a repair response in biopsies collected from painful locations in athletes with MTSS, indicating decreased bone repair activity [13]. Running on hills and uneven surfaces should be avoided, but wearing the right shoes is a crucial tool in treating pes cavus, pes planus, and limiting rearfoot valgus [14]. Arch supports are often recommended to improve lower extremity biomechanics, neuromuscular coordination, muscle fatigue, and pressure distribution in the foot, hence reducing the risk of overuse injuries. Therefore, utilizing an arch support orthosis while running may be a useful method for returning the foot's pressure distribution pattern to normal [15]. Lower-limb muscular stretching has never been shown to prevent MTSS [16]. While flexibility and strength training should be started to correct any muscular imbalances, non-steroidal anti-inflammatory medicines (NSAIDs) can also be used to treat MTSS [14]. Therefore, the purpose of this study was to assess the prevalence, risk factors, and their association with the development of MTSS, as well as the effective treatments that reduce pain and improve functions among the Saudi general population.

## Materials and methods

Study design

The present cross-sectional study was conducted on the general population of Saudi Arabia through an electronic survey over a period of three months. The study sample of 443 people was deemed and considered as estimated by the OpenEpi web tool (Centers for Disease Control and Prevention, Atlanta, GA) with a 95% confidence level. The study included participants from the general population in Saudi Arabia above the age of 18. The data were collected via an online questionnaire. Informed consent was obtained from all participants, and their anonymity and confidentiality were maintained throughout the study and used only for scientific research purposes. The ethical approval of the study was obtained by the chairman of the Institutional Review Board (IRB) Prof. AbdulAziz Al-Akaabba at Imam Mohammad Ibn Saud Islamic University research ethics committee in Riyadh, Saudi Arabia (reference number: 564/2023; dated: 20-3-2024).

A structured self-response questionnaire was given to the participants to assess many variables. The questionnaire evaluated a range of aspects, beginning with information regarding sociodemographics such as age, gender, height, weight, occupation, and residential areas. According to their age, the participants were divided into 18-29, 30-49, and 50 or above. Residential areas were grouped as northern, southern, eastern, western, and middle regions. Moreover, participants were asked generally about their average daily step count, usual walking surfaces, shoe types, continuous running time, whether or not they participate in any sports, and the type of sport they engage in. In addition, participants were asked about their medical condition, including their smoking habits, if they have ever been diagnosed with shin splint/medial tibial stress syndrome, foot or leg deformities "flatfoot or bowlegs'', and a family history of bone diseases or medial tibial stress syndrome, as well as fractures, surgery, or trauma related to their legs. Furthermore, shinbone conditions include pain complaints on the left, right, or both; duration of pain; treatment; preventive care; satisfaction with the management; and recurrence after obtaining the full course of management. The MTSS score consists of 15 questions, including frequency and the content of sporting activities, and the nature and description of pain after sporting activities while standing, walking, performing common daily activities, at rest, and at night [17,18].

Statistical analysis

Microsoft Excel (Microsoft® Corp., Redmond, WA) was used for collecting, cleaning, and coding the included data, while Statistical Product and Service Solutions (SPSS, version 26; IBM SPSS Statistics for Windows, Armonk, NY) was used for data analysis. Mean and standard deviation were used for ongoing variables, while frequency and percentages were used for the description of categorical variables. Chi-test, t-test, and ANOVA were used when adequate, where all statements were considered significant at a p value lower than 0.05.

## Results

The study encompassed a total of 443 participants from the general population of Saudi Arabia, of which (n = 228, 51.5%) were male and (n = 215, 48.5%) were female. In terms of age distribution, the majority fell within the 18-29 age bracket, constituting (n = 227, 51.2%), followed by aged between 30 and 49 (n = 159, 35.9%) and aged 50 or older (n = 57, 12.9%). The mean height recorded was 165.65 cm (SD = 14.91), with a mean weight of 75.34 kg (SD = 19.01), resulting in an average BMI of 30.33 kg/m² (SD = 48.23). Regarding BMI categorization, 4.5% (n = 20) were underweight, 35.7% (n = 158) were of normal weight, 34.1% (n = 151) were overweight, and 25.7% (n = 114) were classified as obese. Occupationally, the majority of participants identified as students (n = 142, 32.1%), followed by office workers (n = 115, 25.9%) and unemployed individuals (n = 60, 13.5%). Healthcare workers constituted a smaller proportion (n = 13, 2.9%), while professional athletes accounted for only 0.5% (n = 2) of the sample. Additionally, the residential distribution indicated a predominance of participants from the central region (n = 398, 89.8%), with lesser representation from the eastern (n = 15, 3.4%), western (n = 22, 5%), southern (n = 5, 1.1%), and northern regions (n = 3, 0.7%) (Table 1).

**Table 1:** Demographic factors of the participants The data have been presented as count, N (%), mean, and standard deviation. SD: Standard deviation

Demographics		Count	N (%)
Gender	Male	228	51.5%
	Female	215	48.5%
Age	18-29	227	51.2%
	30-49	159	35.9%
	50 or older	57	12.9%
Height (cm)	Mean (SD)	165.65 (14.91)	
Weight (Kg)	Mean (SD)	75.34 (19.01)	
BMI (Kg/m2)	Mean (SD)	30.33 (48.23)	
BMI	Underweight	20	4.5%
	Normal weight	158	35.7%
	Overweight	151	34.1%
	Obese	114	25.7%
Occupation	Student	142	32.1%
	Office worker	115	25.9%
	Healthcare worker	13	2.9%
	Professional athlete	2	0.5%
	Academic	44	10.0%
	Farmer	1	0.2%
	Fieldwork	21	4.7%
	Military	18	4.1%
	Unemployed	60	13.5%
	Housewife	8	1.8%
	Retired	19	4.3%
Residential area	Central region	398	89.8%
	Eastern region	15	3.4%
	Western region	22	5.0%
	Southern region	5	1.1%
	Northern region	3	0.7%

Concerning exercise habits, the majority of participants reported walking less than 5,000 steps per day (n = 245, 55.3%), followed by 29.6% (n = 131) walking between 5,000 and 7,500 steps. Most participants reported walking on even or hard surfaces (n = 365, 82.4%) and wearing specific running shoes (n = 237, 53.5%) during their exercise routines. Furthermore, the majority did not engage in continuous running, with 72% (n = 319) reporting no running activity per day. Among those who did engage in sports, various activities were reported, with high-intensity training (n = 93, 44.1%) and running (n = 42, 19.9%) being the most prevalent (Table 2).

**Table 2:** General information considering exercise practice among the participants The data have been presented as count and N (%).

General Information		Count	N (%)
How many steps do you walk per day?	Less than 5000 steps	245	55.3%
	5000 to 7500 steps	131	29.6%
	7500 to 10000 steps	48	10.8%
	10000 to 12500 steps	17	3.8%
	More than 12500 steps	2	0.5%
Most of the time I walk on:	Uneven surfaces (construction area, hiking)	23	5.2%
	Even or hard surfaces (treadmill, sidewalk or buildings)	365	82.4%
	Customized surfaces (running tracks)	37	8.4%
	Soft surfaces (sand, grass)	18	4.1%
Most of the time I wear:	Specific running shoes	237	53.5%
	Formal/non-specific running shoes	125	28.2%
	Slippers	71	16.0%
	No shoes	10	2.3%
What is your continues running time per day?	No running	319	72.0%
	Less than 30 min	74	16.7%
	30 to 45 min	30	6.8%
	45 to 59 min	9	2.0%
	An hour to an hour and a half	9	2.0%
	More than an hour and a half	2	0.5%
Do you play any kind of sport?	No	232	52.4%
	Yes	211	47.6%
What does the sport you play include?	Jumping (e.g., volleyball)	14	6.6%
	Running (e.g., football)	42	19.9%
	High-intensity training (resistance training)	93	44.1%
	Low-intensity training (swimming)	34	16.1%
	Walking	28	13.3%

Table 3 presents the prevalence of shin splints/MTSS and other associated medical conditions among the participants. The majority (n = 435, 98.2%) reported no prior diagnosis of shin splints/MTSS, while only 1.8% (n = 8) confirmed such a diagnosis. Regarding family history, 96.2% (n = 426) stated no familial occurrence of bone diseases, or MTSS, with 3.8% (n = 17) reporting a positive family history. Moreover, 91% (n = 403) denied any previous diagnosis of foot or leg deformities such as flatfoot or bowlegs, while 9% (n = 40) acknowledged such conditions. Concerning tobacco use, the majority (n = 342, 77.2%) reported never smoking, while 16.7% (n = 74) were current smokers, and 6.1% (n = 27) were former smokers. Additionally, 89.2% (n = 395) reported no history of leg-related fractures, surgeries, or traumas, while 10.8% (n = 48) confirmed experiencing such incidents, with the distribution indicating involvement of both legs (n = 13, 27.1%), the left leg (n = 19, 39.6%), and the right leg (n = 16, 33.3%).

**Table 3:** The prevalence of shin splint/medial tibial stress syndrome and other medical conditions The data have been presented as count and N (%).

Medical Condition		Count	N (%)
Have you ever been diagnosed with shin splint/medial tibial stress syndrome?	No	435	98.2%
	Yes	8	1.8%
Is there any family history of bone diseases or medial tibial stress syndrome?	No	426	96.2%
	Yes	17	3.8%
Have you ever been diagnosed with any foot or leg deformities (flatfoot or bowlegs)?	No	403	91.0%
	Yes	40	9.0%
Have you ever smoked tobacco?	Never	342	77.2%
	Yes, current	74	16.7%
	Yes, but smoked in the past	27	6.1%
Have you had any fracture, surgery or trauma related to your leg?	No	395	89.2%
	Yes	48	10.8%
In which leg have you had a fracture, surgery or trauma?	Both	13	27.1%
	Left	19	39.6%
	Right	16	33.3%

Table 4 elucidates the prevalence and characteristics of shin pain among the participants. Of the total, 21% (n = 93) reported experiencing shin pain currently or previously, with complaints in both shins (n = 50, 53.8%), the left shin only (n = 25, 26.9%), and the right shin only (n = 18, 19.4%). Among those experiencing pain, the majority reported the most complaints in the left shin (n = 51, 54.8%). Pain distribution varied, with some experiencing pain predominantly in the high (n = 22, 23.7%), medium (n = 34, 36.6%), or lower (n = 12, 12.9%) parts of the shin, while others reported pain across the entire shin (n = 25, 26.9%). The duration of pain varied, with 21.5% (n = 20) reporting pain lasting less than six weeks and 78.5% (n = 73) experiencing pain for longer durations. Moreover, 68.8% (n = 64) of participants had not received treatment for their pain, with 31.2% (n = 29) having sought treatment. Of those treated, 58.6% (n = 17) expressed satisfaction with the management received, while 41.4% (n = 12) were not satisfied. Furthermore, 79.3% (n = 23) reported a recurrence of shin pain after completing the management course.

**Table 4:** Prevalence of shin pain and its characteristics The data have been presented as count and N (%).

Shinbone Pain		Count	N (%)
Presence of shin pain now/previously?	No	350	79.0%
	Yes	93	21.0%
I have complaints in:	Both shins	50	53.8%
	Only left shin	25	26.9%
	Only right shin	18	19.4%
I have most complaints in:	Left shin	51	54.8%
	Right shin	42	45.2%
I have the pain mostly in:	High part of shin	22	23.7%
	Medium part of shin	34	36.6%
	Lower part of shin	12	12.9%
	Complete	25	26.9%
I have the pain for:	Less than 6 weeks	20	21.5%
	Less than 6 weeks	73	78.5%
Have you actually experienced treatment for the pain?	No	64	68.8%
	Yes	29	31.2%
Are you satisfied with the management you have experienced?	No	12	41.4%
	Yes	17	58.6%
Have the symptoms of shin pain reoccurred after the management course?	No	6	20.7%
	Yes	23	79.3%

**Figure 1:**
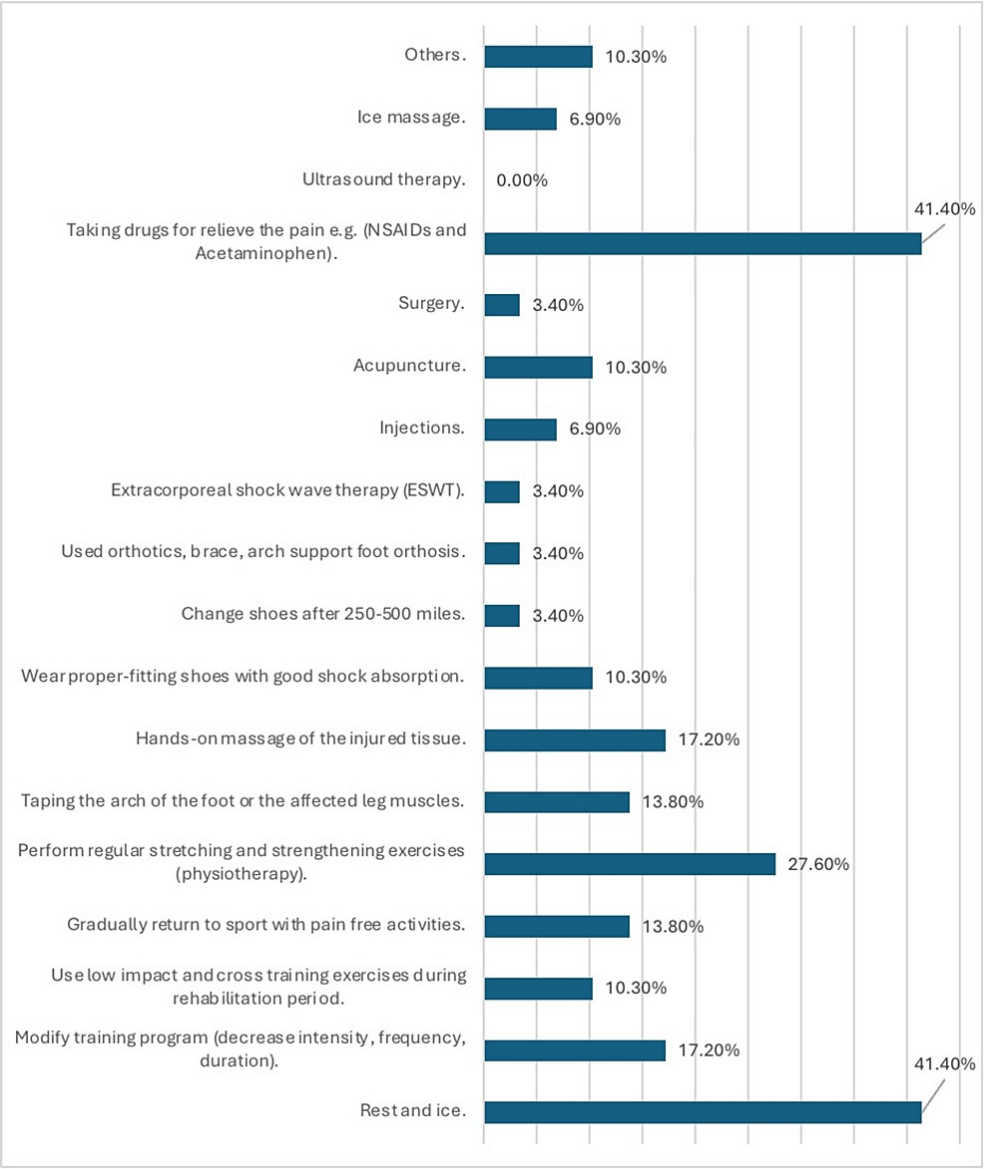
Treatment strategies reported by 29 participants The data have been presented as N (%).

The treatment strategies reported by the 29 participants who sought treatment for shin pain are summarized in Figure 1. Common strategies included rest and ice (n = 12, 41.4%), taking pain-relieving drugs (n = 12, 41.4%), and regular stretching and strengthening exercises (n = 8, 27.6%).

**Figure 2:**
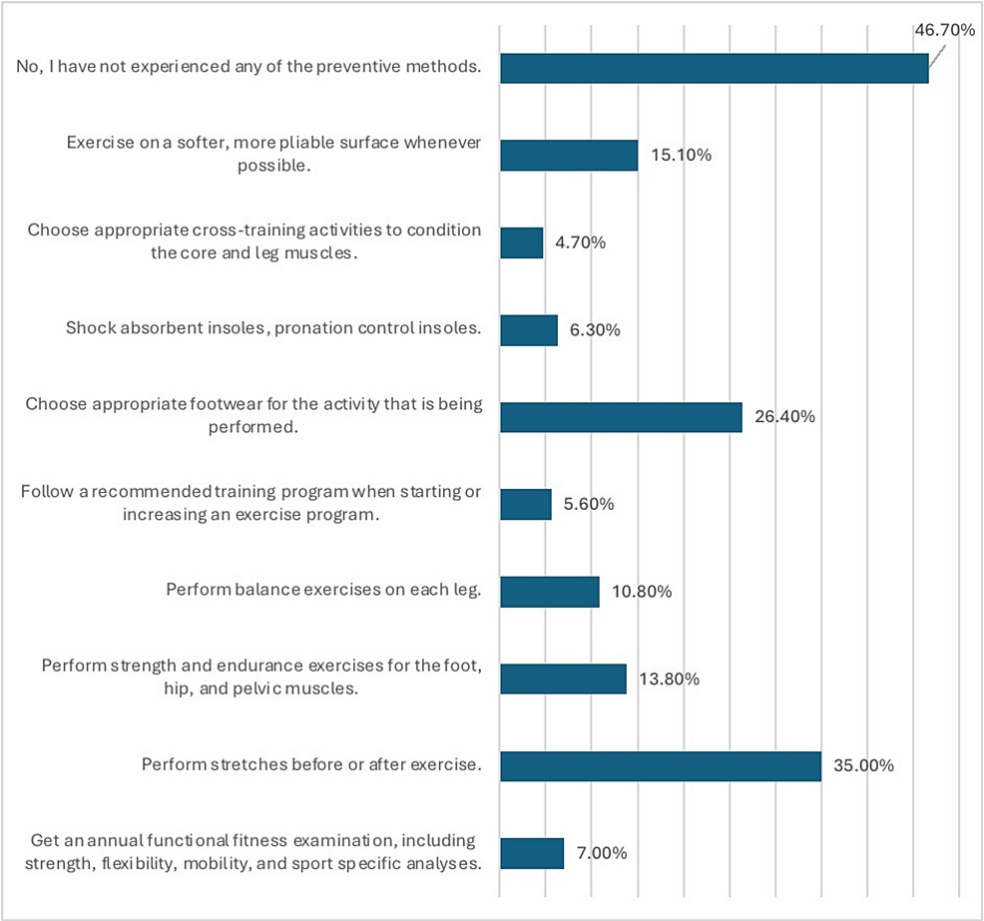
Preventive methods for shin pain among the participants (N-443) The data have been presented as N (%).

Figure 2 outlines the preventive methods for shin pain among the participants. Notable preventive measures included performing stretches before or after exercise (n = 155, 35%), choosing appropriate footwear (26.4%), and exercising on softer surfaces when possible (n = 67, 15.1%). However, 46.7% (n = 207) of the participants reported not having experienced any of the preventive methods.

The assessment of participants' experiences regarding shin pain and its impact on their sporting activities revealed several significant findings. Presently, the majority (n = 381, 86%) reported being able to perform all of their usual sporting activities, while a smaller proportion reported experiencing limitations due to shin pain, with 5.9% (n = 26) being forced to reduce their activities and 1.6% (n = 7) unable to engage in any sporting activity. Additionally, participants indicated varying degrees of adjustment to their sporting activities due to shin pain, with 72.7% (n = 322) reporting no adjustments and smaller percentages (n = 45, 10.2%) reporting slight, substantial (n = 28, 6.3%), or complete adjustments (n = 22, 5%). Notably, 5.9% (n = 26) reported being unable to participate in any sporting activities due to shinbone pain. Regarding the onset and persistence of pain during sporting activities, the majority reported experiencing no pain during activities (n = 345, 77.9%), while 16.9% (n = 75) reported some pain, and 1.8% (n = 8) reported a lot of pain. When the pain did occur, it typically manifested after 15 minutes of starting the activity for 13.5% (n = 60) of participants, immediately for 3.4% (n = 15), and within the first 15 minutes for 4.5% (n = 20). However, a sizable proportion (n = 15, 3.4%) reported being unable to engage in any sporting activity due to shin pain. After engaging in sporting activities, 71.6% (n = 317) reported experiencing no pain, while 12.6% (n = 56) reported pain disappearing within 12 hours and 7% (n = 31) within 12 hours to two days. Conversely, 4.7% (n = 21) reported pain persisting for longer than two days, with 4.1% (n = 18) unable to engage in any further activities due to shin pain. Similar patterns were observed for pain experienced while standing, walking, and going up or down stairs, with varying degrees of pain reported, and a small proportion reporting being unable to engage in activities due to pain. At rest, the majority reported no pain in the shin (n = 372, 84%), while 9.7% (n = 43) reported sensitivity, and 5.4% (n = 24) reported pain. During the night, 82.8% (n = 367) reported no pain, while 9.7% (n = 43) reported occasional sensitivity, and smaller percentages reported waking up due to pain (n = 25, 5.6%) or experiencing sleep disturbances due to pain (n = 8, 1.8%). Additionally, participants reported varying degrees of pain when touching their shin, with the majority (n = 365, 82.4%) experiencing no pain, while smaller percentages reported pain when bumping (n = 31, 7%), pressing (n = 34, 7.7%), or rubbing, pressing, and bumping their shin (n = 13, 2.9%). Overall, the mean MTSS score was relatively low, indicating mild symptoms on average among participants (mean = 1.09, SD = 1.98) (Supplement Table 7).

Table 5 presents the association between the prevalence of shin splint/MTSS and demographic factors among participants. Among gender groups, a slightly higher proportion of males (n = 5, 2.2%) reported a diagnosis of shin splint/MTSS compared to females (n = 3, 1.4%), although the difference was not statistically significant (p = 0.529). Similarly, no significant associations were observed between age groups, BMI categories, occupation, residential areas, or daily step counts and the prevalence of shin splint/MTSS (all p-values > 0.05). However, significant associations were found between the prevalence of shin splint/MTSS and certain exercise-related factors. Participants who primarily walked on uneven surfaces, such as construction areas or hiking trails, exhibited a higher prevalence of shin splint/MTSS (n = 2, 8.7%) compared to those who walked on other surfaces (p = 0.017). Similarly, participants who engaged in continuous running for longer durations per day showed a higher prevalence of shin splint/MTSS, with significant differences observed between different running time categories (p = 0.000). Furthermore, participants who reported engaging in any kind of sport had a significantly higher prevalence of shin splint/MTSS (n = 7, 3.3%) compared to those who did not participate in sports (n = 1, 0.4%) (p = 0.023).

**Table 5:** The association between the prevalence of shin splint/medial tibial stress syndrome and demographic factors The data have been presented as count, N (%), and p-value. *p value <0.05 is considered significant.

Demographics		Have you ever been diagnosed with shin splint/medial tibial stress syndrome?				
		No		Yes		
		Count	N (%)	Count	N (%)	Overall p-value
Gender	Male	223	97.8%	5	2.2%	0.529
	Female	212	98.6%	3	1.4%	
Age	18-29	224	98.7%	3	1.3%	0.686
	30-49	155	97.5%	4	2.5%	
	50 or older	56	98.2%	1	1.8%	
BMI	Underweight	20	100.0%	0	0.0%	0.496
	Normal weight	154	97.5%	4	2.5%	
	Overweight	150	99.3%	1	0.7%	
	Obese	111	97.4%	3	2.6%	
Occupation	Student	141	99.3%	1	0.7%	0.088
	Office worker	113	98.3%	2	1.7%	
	Healthcare worker	11	84.6%	2	15.4%	
	Professional athlete	2	100.0%	0	0.0%	
	Academic	43	97.7%	1	2.3%	
	Farmer	1	100.0%	0	0.0%	
	Fieldwork	20	95.2%	1	4.8%	
	Military	18	100.0%	0	0.0%	
	Unemployed	59	98.7%	1	1.6%	
	Housewife	8	100.0%	0	0.0%	
	Retired	19	100.0%	0	0.0%	
Residential area	Central region	391	98.2%	7	1.8%	0.635
	Eastern region	14	93.3%	1	6.7%	
	Western region	22	100.0%	0	0.0%	
	Southern region	5	100.0%	0	0.0%	
	Northern region	3	100.0%	0	0.0%	
How many steps do you walk per day?	Less than 5000 steps	242	98.8%	3	1.2%	0.682
	5000 to 7500 steps	128	97.7%	3	2.3%	
	7500 to 10000 steps	47	97.9%	1	2.1%	
	10000 to 12500 steps	16	94.1%	1	5.9%	
	More than 12500 steps	2	100.0%	0	0.0%	
Most of the time I walk on:	Uneven surfaces (construction area, hiking)	21	91.3%	2	8.7%	0.017*
	Even or hard surfaces (treadmill, sidewalk or buildings)	361	98.9%	4	1.1%	
	Customized surfaces (running tracks)	35	94.6%	2	5.4%	
	Soft surfaces (sand, grass)	18	100.0%	0	0.0%	
Most of the time I wear:	Specific running shoes	233	98.3%	4	1.7%	0.916
	Formal/non-specific running shoes	122	97.6%	3	2.4%	
	Slippers	70	98.6%	1	1.4%	
	No shoes	10	100.0%	0	0.0%	
What is your continues running time per day?	No running	318	99.7%	1	0.3%	0.000*
	Less than 30 min	72	97.3%	2	2.7%	
	30 to 45 min	29	96.7%	1	3.3%	
	45 to 59 min	7	77.8%	2	22.2%	
	An hour to an hour and a half	8	88.9%	1	11.1%	
	More than an hour and a half	1	50.0%	1	50.0%	
Do you play any kind of sport?	No	231	99.6%	1	0.4%	0.023*
	Yes	204	96.7%	7	3.3%	

Table 6 presents the association between the MTSS score and demographic factors. Significant associations were found between the MTSS score and several demographic variables. Specifically, males exhibited a lower mean score (0.83) compared to females (1.37), indicating less severe symptoms among males (p = 0.004). Age was also significantly associated with the MTSS score, with older participants demonstrating higher mean scores compared to younger age groups (p = 0.000). Similarly, BMI categories showed significant associations, with higher mean scores observed among participants classified as obese (1.76) compared to those classified as underweight or of normal weight (p = 0.000). Occupation was also significantly associated with the MTSS score, with variations observed among different occupational groups (p = 0.024). Additionally, significant associations were found between the MTSS score and certain exercise-related factors, including walking surface, type of footwear worn, and engagement in sports activities (all p-values < 0.05). Participants diagnosed with a shin splint/MTSS had a substantially higher mean score (3.25) compared to those without the diagnosis (1.05) (p = 0.002), indicating more severe symptoms among individuals with a previous diagnosis.

**Table 6:** The association between the medial tibial stress syndrome score and demographic factors The data have been presented as mean, standard deviation, and p-value. *p value <0.05 is considered significant.

Demographics		Medial Tibial Stress Syndrome Score		
		Mean	Standard Deviation	Overall p-value
Gender	Male	0.83	1.65	0.004*
	Female	1.37	2.26	
Age	18-29	0.76	1.57	0.000*
	30-49	1.22	2.04	
	50 or older	2.07	2.82	
BMI	Underweight	1.20	2.53	0.000*
	Normal weight	0.59	1.30	
	Over-weight	1.10	1.94	
	Obese	1.76	2.48	
Occupation	Student	0.77	1.74	0.024*
	Office worker	0.97	1.72	
	Healthcare worker	1.23	2.49	
	Professional athlete	1.00	1.41	
	Academic	1.48	1.98	
	Farmer	0.00	0.00	
	Fieldwork	0.43	0.81	
	Military	1.11	1.53	
	Unemployed	1.57	2.64	
	Housewife	3.00	3.89	
	Retired	1.58	2.22	
Residential area	Central region	1.06	1.98	0.696
	Eastern region	1.33	1.45	
	Western region	1.64	2.52	
	Southern region	0.80	1.30	
	Northern region	0.67	1.15	
How many steps do you walk per day?	Less than 5000 steps	1.13	2.09	0.717
	5000 to 7500 steps	1.02	1.77	
	7500 to 10000 steps	0.98	1.96	
	10000 to 12500 steps	1.59	2.29	
	More than 12500 steps	0.00	0.00	
Most of the time I walk on:	Uneven surfaces (construction area, hiking)	2.09	2.64	0.040*
	Even or hard surfaces (treadmill, sidewalk or buildings)	1.00	1.88	
	Customized surfaces (running tracks)	1.49	2.51	
	Soft surfaces (sand, grass)	0.83	1.54	
Most of the time I wear:	Specific running shoes	0.88	1.74	0.030*
	Formal/non-specific running shoes	1.26	1.95	
	Slippers	1.59	2.69	
	No shoes	0.50	1.27	
What is your continues running time per day?	No running	1.14	2.03	0.377
	Less than 30 min	0.78	1.74	
	30 to 45 min	1.10	2.02	
	45 to 59 min	0.67	1.66	
	An hour to an hour and a half	2.22	2.28	
	More than an hour and a half	1.00	1.41	
Do you play any kind of sport?	No	1.30	2.21	0.023*
	Yes	0.87	1.68	
Have you ever been diagnosed with shin splint/medial tibial stress syndrome?	No	1.05	1.93	0.002*
	Yes	3.25	3.41	

## Discussion

The present study aimed to assess the prevalence, risk factors, and management strategies associated with MTSS, commonly known as shin splints, among the general population of Saudi Arabia. Our findings shed light on various demographic and exercise-related factors that contribute to the prevalence and severity of MTSS, providing valuable insights for preventive and management approaches.

The prevalence of MTSS in our study population was relatively low, with only 1.8% (n = 8) of participants reporting a previous diagnosis. This finding aligns with previous research indicating a relatively low prevalence of MTSS in the general population [8,19]. However, it is essential to note that the prevalence may vary across different populations and demographic groups. However, our results are lower than those reported in other studies among specific groups, particularly those who practice regular exercises, including a study by Bliekendaal et al. among first-year Dutch physical education teacher education (PETE) students, in which the prevalence of MTSS was 25% (n = 55) during the follow-up period [20]. Verrelst et al. reported a 26% (n = 21) incidence of MTSS among female PETE students over a 29-week follow-up period [21]. Sharma et al. observed an incidence of 7.9% (n = 37) among male recruits (n = 468) during a 26-week training period [22]. Rauh et al. found a 7.2% (n = 54) incidence in female Marine Corps recruits over a 13-week training period [23]. Yates et al. documented an incidence of 35% (n = 40) among 124 naval recruits, with rates of 67.7% (n = 84) for men and 32.3% (n = 40) for women, during a 10-week basic training program [24]. Bennett et al. studied 125 high school cross-country runners, noting a 12% (n = 15) incidence during an eight-week training program [25].

Our study did not find a significant association between gender, age, BMI, occupation, residential area, or daily step count and the prevalence of MTSS. This finding contradicts some previous studies that have suggested an association between certain demographic factors and the risk of MTSS. For example, a study by Yates et al. found that female gender and higher BMI were significant risk factors for developing MTSS [24]. In addition, Bliekendaal et al. showed more women developed MTSS during the follow-up period (39%) than men (21%) [20]. The lack of significant associations in our study may be attributed to the relatively small sample size or differences in population characteristics.

Interestingly, our study identified several exercise-related factors that were significantly associated with the prevalence of MTSS. Participants who primarily walked on uneven surfaces, such as construction areas or hiking trails, exhibited a higher prevalence of MTSS compared to those who walked on other surfaces. This finding is consistent with previous research highlighting the importance of training surface characteristics in the development of MTSS. Pohl et al. demonstrated that running on hard surfaces, such as concrete or asphalt, significantly increased the risk of developing MTSS compared to running on softer surfaces, such as grass or sand [26].

Moreover, participants who engaged in continuous running for longer durations per day showed a higher prevalence of MTSS. This finding underscores the potential role of training volume and intensity in the development of MTSS [8]. Several studies have suggested that excessive or rapid increases in training volume or intensity may overload the tibia and surrounding muscles, leading to the development of MTSS [13]. Therefore, gradual progression and appropriate training modifications are essential for reducing the risk of MTSS among athletes and active individuals.

Additionally, participants who reported engaging in any kind of sport had a significantly higher prevalence of MTSS compared to those who did not participate in sports. This finding highlights the impact of sports participation on the risk of MTSS, emphasizing the importance of sport-specific biomechanical factors and training techniques in MTSS development. Previous research has identified specific sports, such as running, basketball, and soccer, as high-risk activities for MTSS due to repetitive impact loading and biomechanical stress on the tibia [27].

Regarding the severity of MTSS symptoms, our study found significant associations between demographic factors and the MTSS score. Males exhibited less severe symptoms compared to females, while older participants and those classified as obese demonstrated higher MTSS scores. These findings are consistent with previous research suggesting that female gender, older age, and a higher BMI are associated with more severe MTSS symptoms [8,28,29]. The observed differences in symptom severity may be attributed to variations in biomechanical factors, muscle strength, and bone density among different demographic groups.

Furthermore, certain exercise-related factors, such as walking surface, type of footwear worn, and engagement in sports activities, were significantly associated with the MTSS score. Participants diagnosed with MTSS had substantially higher MTSS scores compared to those without the diagnosis, indicating more severe symptoms among individuals with a previous diagnosis. These findings emphasize the importance of identifying and modifying modifiable risk factors and implementing appropriate management strategies to alleviate MTSS symptoms and prevent recurrence.

For treatment options, our study revealed a variety of strategies employed by participants to alleviate the symptoms of MTSS. Among the reported treatments, rest and ice application emerged as commonly utilized approaches, aligning with established recommendations for the initial management of MTSS symptoms [30]. Furthermore, participants frequently engaged in physiotherapy involving regular stretching and strengthening exercises, which have shown efficacy in improving lower limb biomechanics and reducing pain associated with MTSS [13]. Additionally, the use of proper-fitting shoes with adequate shock absorption and the administration of pain-relieving drugs, such as NSAIDs and acetaminophen, were reported by participants as effective strategies for symptom management, consistent with current clinical guidelines [25]. However, it is imperative to acknowledge the individual variability in treatment responses and the importance of personalized approaches tailored to each patient's specific needs and circumstances.

On the other hand, preventive measures taken by participants underscored the importance of addressing modifiable risk factors to mitigate the occurrence and recurrence of MTSS. Our findings indicated that incorporating pre- and post-exercise stretching routines was a commonly adopted practice among participants, aligning with evidence suggesting the role of flexibility exercises in reducing the risk of MTSS [31]. Moreover, participants emphasized the significance of choosing appropriate footwear for their activities, including shock-absorbent insoles and pronation control features, to minimize excessive biomechanical stress on the lower limbs during physical exertion [32]. Furthermore, adhering to recommended training programs and gradually increasing exercise intensity emerged as crucial strategies to prevent overuse injuries such as MTSS, highlighting the importance of structured training regimens and progressive loading protocols in injury prevention [13]. These findings emphasize the need for comprehensive injury prevention programs that encompass education on proper training techniques, footwear selection, and biomechanical principles to optimize musculoskeletal health and reduce the burden of MTSS among athletes and active individuals.

Despite providing valuable insights into the prevalence, risk factors, and management strategies associated with MTSS among the Saudi Arabian population, several limitations should be considered when interpreting the study findings. Firstly, the cross-sectional design of the study limits the establishment of causal relationships between demographic or exercise-related factors and MTSS prevalence or severity. Additionally, reliance on self-reported data, including diagnosis of MTSS and treatment outcomes, may introduce recall bias and affect the accuracy of reported findings. Moreover, the study's reliance on an electronic survey administered to the general population may lead to sampling bias, potentially excluding individuals without internet access or those less inclined to participate in online surveys. In addition, the absence of a detailed description regarding the treatments used in the study, including information on the frequency and duration, poses a limitation. The lack of such details may interfere with the effectiveness of specific treatment approaches for MTSS, which vary depending on factors such as the frequency and duration of interventions. Therefore, future research utilizing longitudinal designs, objective measures of MTSS diagnosis and treatment outcomes, specific details on treatment, and larger more diverse samples is warranted to address these limitations and provide a more comprehensive understanding of MTSS epidemiology and management strategies to facilitate better interpretation of the research.

## Conclusions

In conclusion, our study provides valuable insights into the prevalence, risk factors, and management strategies associated with MTSS among the general population of Saudi Arabia. The findings underscore the complex interplay of demographic and exercise-related factors in the development and severity of MTSS and highlight the importance of targeted preventive and management approaches. Future research should focus on further elucidating the underlying mechanisms of MTSS and evaluating the effectiveness of multifaceted interventions in reducing the burden of this common lower limb overuse injury.

## Appendices

**Table 7:** The medial tibial stress syndrome score The data have been presented as count and N (%). MTSS = Medial tibial stress syndrome

MTSS Score		Count	N (%)
Presently	I perform all of my usual sporting activities.	381	86.0%
	I am forced to do less of my usual sporting activities due to pain in my shin.	26	5.9%
	I am forced to do alternative sporting activities only due to pain in my shin.	7	1.6%
	I cannot do any sporting activity due to pain in my shin.	29	6.5%
The frequency of your sporting activities:	I have not reduced the frequency of my sporting activities.	283	63.9%
	I have reduced the frequency of my sporting activities by 1-25% a week.	56	12.6%
	I have reduced the frequency of my sporting activities by 26-50% a week.	30	6.8%
	I have reduced the frequency of my sporting activities by 51-75% a week.	22	5.0%
	I have reduced the frequency of my sporting activities by 76-100% a week.	52	11.7%
The content of your sporting activities	I have not adjusted my sporting activity.	322	72.7%
	I have adjusted my sporting activity slightly.	45	10.2%
	I have adjusted my sporting activities substantially	28	6.3%
	I have adjusted the majority of my sporting activities	22	5.0%
	I cannot do any sporting activities due to my shinbone pain	26	5.9%
While performing sporting activities	I have no pain in my shin.	345	77.9%
	I have some pain in my shin.	75	16.9%
	I have a lot of pain in my shin.	8	1.8%
	I cannot do any sporting activity, due to my shin pain.	15	3.4%
How long, after you have started a sporting activity, do you feel the pain in your shin?	I have no pain during sporting activities.	333	75.2%
	After 15 minutes, after I have started.	60	13.5%
	Within the first 15 minutes after I have started.	20	4.5%
	Immediately after I have started.	15	3.4%
	I cannot do any sporting activity do you to my shinbone pain.	15	3.4%
In the case of pain being present during your sporting activities, and you continue the activity, what happens to your pain?	I have no pain during sporting activities.	313	70.7%
	The pain decreases.	45	10.2%
	The pain remains unchanged.	41	9.3%
	The pain increases.	28	6.3%
	I cannot do any sporting activities due to my shinbone pain.	16	3.6%
If you feel pain in your shin when starting your sporting activity, and you continue the activity, what happens to your pain?	I have no pain during sporting activities.	316	71.3%
	The pain disappears within 10 minutes.	31	7.0%
	The pain disappears after 10 minutes.	31	7.0%
	The pain does not disappear.	46	10.4%
	I cannot do any sporting activities due to my shinbone pain.	19	4.3%
After sporting activities	I have no pain.	317	71.6%
	The pain disappears within 12 hours.	56	12.6%
	The pain disappears between 12 hours to 2 days.	31	7.0%
	The pain remains present for longer than 2 days.	21	4.7%
	I cannot do any sporting activity due to my shinbone pain.	18	4.1%
While standing	I have no pain in my shin.	304	68.6%
	I have some pain in my shin.	110	24.8%
	I have a lot of pain in my shin.	23	5.2%
	I cannot do any sporting activity, due to my shin pain.	6	1.4%
While walking	I have no pain in my shin.	337	76.1%
	I have some pain in my shin.	90	20.3%
	I have a lot of pain in my shin.	13	2.9%
	I cannot do any sporting activity, due to my shin pain.	3	0.7%
While going up or down stairs:	I have no pain in my shin.	320	72.2%
	I have some pain in my shin.	93	21.0%
	I have a lot of pain in my shin.	21	4.7%
	I cannot do any sporting activity, due to my shin pain.	9	2.0%
While performing common daily activities:	I have no pain in my shin.	353	79.7%
	I have some pain in my shin.	76	17.2%
	I have a lot of pain in my shin.	7	1.6%
	I cannot do any sporting activity, due to my shin pain.	7	1.6%
At rest (sitting or lying down), my shin is:	Not painful.	372	84.0%
	Sensitive.	43	9.7%
	Painful.	24	5.4%
	Very painful.	4	0.9%
At night:	I have no pain.	367	82.8%
	My shin is sometimes sensitive.	43	9.7%
	I wake up sometimes because of the pain in my shin.	25	5.6%
	I cannot sleep due to the pain in my shin for parts of the night.	8	1.8%
Pain while touching:	I have no pain when touching my shin.	365	82.4%
	I have pain when I bump my shin.	31	7.0%
	I have pain when I press and when I bump my shin.	34	7.7%
	I have pain when I rub, press and when I bump my shin.	13	2.9%
